# Pulmonary artery remodeling and wall shear stress in repaired tetralogy of fallot: insights from 4D flow MRI before and after transcatheter pulmonary valve implantation

**DOI:** 10.1007/s10554-026-03726-5

**Published:** 2026-05-11

**Authors:** Hirozumi Mori, Michinobu Nagao, Tomohito Kogure, Seiji Asagai, Kei Inai, Akihiro Inoue, Atsushi Yamamoto, Junichi Yamaguchi, Takeshi Shinkawa, Masahiro Jinzaki

**Affiliations:** 1 Department of Radiology, Keio University School of Medicine, 35 Shinanomachi, Shinjuku-ku, Tokyo, 160-8582 Japan; 2 Department of Diagnostic Imaging and Nuclear Medicine, Tokyo Women’s Medical University, 8-1 Kawada-cho, Shinjuku-ku, Tokyo, Tokyo, 162-8666 Japan; 3 Department of Cardiology, Tokyo Women’s Medical University, 8-1 Kawada-cho, Shinjuku-ku, Tokyo, Tokyo, 162-8666 Japan; 4 Department of Pediatric Cardiology, Tokyo Women’s Medical University, Tokyo, 8-1 Kawada-cho, Shinjuku-ku, Tokyo, 162-8666 Japan; 5 Department of Cardiovascular Surgery, Tokyo Women’s Medical University, Tokyo, 8-1 Kawada-cho, Shinjuku-ku, Tokyo, 162-8666 Japan

**Keywords:** Tetralogy of fallot, Pulmonary regurgitation, Right ventricular remodeling, Transcatheter pulmonary valve implantation, 4D Flow MRI, Wall shear stress

## Abstract

To investigate hemodynamic factors associated with pulmonary artery (PA) remodeling in repaired tetralogy of Fallot (rTOF), focusing on wall shear stress (WSS) and pulmonary regurgitation (PR), and to assess pre- and post-procedural changes following transcatheter pulmonary valve implantation (TPVI) using 4D flow MRI. Thirty rTOF patients undergoing TPVI were analyzed using pre- and 3-month post-procedural 4D flow MRI. Regurgitant fraction (RF-4D), ventricular parameters, and WSS along the main pulmonary artery (MPA) were quantified. Pre-TPVI, RF-4D correlated with MPA dilation (*r* = 0.567, *p* = 0.001), whereas no association was observed with mean pulmonary arterial pressure. Both systolic and diastolic WSS showed significant negative correlations with MPA area. Multivariable analysis demonstrated that RF-4D (β = 11.52, *p* < 0.001) and diastolic WSS (β = -0.54, *p* = 0.003) were independently associated with MPA dilation, while age was not significant. Post-TPVI, RF-4D decreased markedly with right ventricular reverse remodeling. Early diastolic WSS significantly decreased, while systolic WSS remained unchanged, resulting in resolution of the biphasic WSS pattern. In rTOF, pulmonary artery dilation was more strongly associated with pulmonary regurgitation-derived flow metrics than with pressure, suggesting a dominant role of flow-related hemodynamic stress. Diastolic WSS decreased after TPVI in parallel with reduction of PR and may reflect the stage of vascular remodeling. These findings suggest that WSS may serve as a mechanistic marker of pulmonary artery remodeling and may provide additional value for risk stratification in patients with progressive pulmonary regurgitation and PA dilation.

## Introduction

In patients with tetralogy of Fallot (TOF), pulmonary regurgitation is known to worsen after right ventricular outflow tract (RVOT) repair, potentially contributing to long-term clinical deterioration, including heart failure and arrhythmias in some patients [1, 2]. 

Although there are reports on the mechanisms worsening pulmonary regurgitation, such as vulnerability of the repair site and the effects of right ventricular dilation, in‑vivo studies investigating it from a hemodynamic or fluid‑mechanical perspective remain scarce [3]. 

4D flow MRI can measure wall shear stress (WSS) in blood vessels by providing three-dimensional velocity and directional information of blood flow throughout a volume, acquired over multiple phases of the cardiac cycle [4, 5]. Furthermore, the three-dimensional volumetric data allow visualization and quantification of the pulmonary artery anatomy, enabling an integrated hemodynamic assessment of the relationship between pulmonary regurgitation and pulmonary artery remodeling.

This study aims to assess the factors that cause pulmonary artery remodeling, particularly focusing on the impact of wall shear stress, a key mechanical force influencing endothelial mechanotransduction, a process by which endothelial cells sense mechanical forces, such as shear stress and convert them into biological responses, including nitric oxide production, inflammation, and vascular remodeling [6–8].

**Fig. 1:**
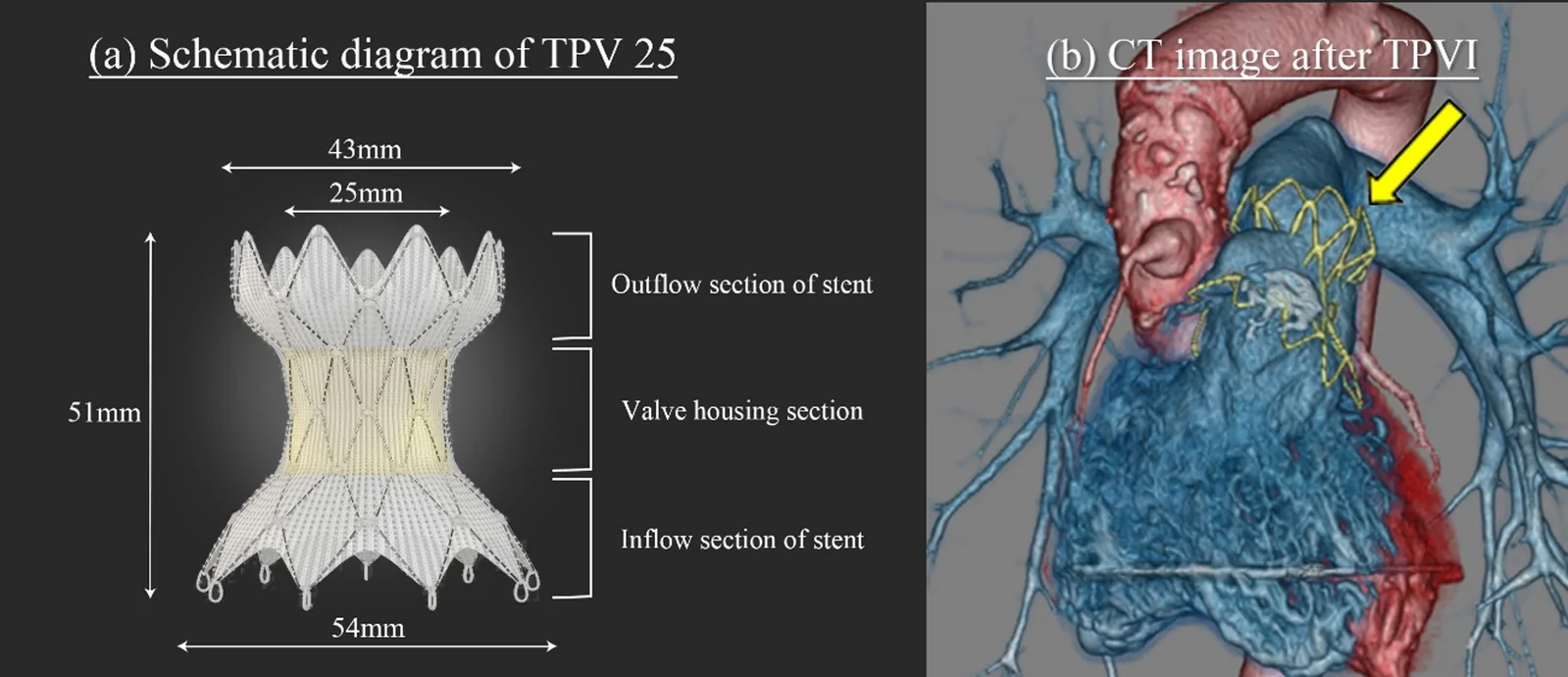
Medtronic’s Harmony TPV 25 (reproduced with permission from MEDTRONIC JAPAN CO., LTD.). (**a**) The bioprosthetic valve is attached in the valve housing section. (**b**) Post-TPVI contrast-enhanced CT image showing the Harmony 25 (arrow) positioned in the pulmonary artery. The outflow section of the stent is positioned distally in the pulmonary artery. The inflow section of the stent is placed in the right ventricular outflow tract

We also assessed the changes in pulmonary artery WSS following transcatheter pulmonary valve implantation (TPVI)—which has recently become more widely performed as an alternative to surgical pulmonary valve replacement—and how these changes relate to the reduction in pulmonary regurgitation [9, 10]. In Japan, Medtronic’s Harmony TPV25, which was FDA-approved in 2021 as a TPVI device, has been insurance listed and is used for treatment (Fig. 1).

To the best of our knowledge, no existing reports exist on pre- and post-TPVI observations using 4D flow, a novel imaging technique.

## Methods

### Study design

This single-center retrospective study included patients who underwent TPVI using Harmony TPV 25 between 2023 and 2024. The inclusion criteria were (1) repaired TOF and (2) completion of CMR, including 4D Flow, both pre- and at 3 months post-TPVI. Patients without preoperative 4D flow data were excluded, as they had been referred from other hospitals and underwent TPVI based on the results of prior conventional CMR examinations.

### CMR and 4D flow acquisition

Pre- and post-TPVI MRI examinations were performed using a 3.0-T whole-body scanner (Ingenia 3 Tesla; Philips Healthcare, Best, the Netherlands) equipped with a dual-source parallel radiofrequency transmitter and a 32-element D-shaped torso coil for signal reception.

The CMR protocol included cine balanced steady-state free precession (bSSFP) imaging to assess ventricular size and function (end diastolic volume index [EDVi] and end systolic volume index [ESVi]), ejection fraction [EF] and cardiac output [CO]), and 4D flow MRI for pulmonary flow assessment.

Cine bSSFP images were acquired using contiguous short-axis stacks covering both ventricles. Imaging parameters were as follows: field of view 360 × 360 mm²; echo time/repetition time (TE/TR) 2/3 ms; flip angle 50°; matrix size 192 × 141; slice thickness 10 mm; and an acquired temporal resolution of 30–51 ms depending on heart rate with 20 frames per cardiac cycle using retrospective electrocardiographic gating. Chamber views including vertical long-axis 2-chamber, horizontal long-axis 2-chamber, 4-chamber, and contiguous short-axis slices were obtained. Ventricular volumes were measured from the short-axis cine stack.

For 4D flow MRI, time-resolved flow-sensitive three-dimensional gradient-echo imaging with echo-planar imaging (EPI) readout was performed. Imaging parameters were as follows: velocity encoding (VENC) 200 cm/s; field of view 360 × 360 mm²; matrix size 128 × 256; sensitivity encoding (SENSE) factor 3; EPI factor 3; TE 3.2 ms; TR 6.2 ms; flip angle 12°; turbo field echo (TFE) factor 4; and an acquired temporal resolution of 64–101 ms depending on heart rate with 10 frames per cardiac cycle. Imaging was performed in the coronal orientation with retrospective cardiac triggering during free breathing. The acquired spatial resolution was 1.97 × 3.28 × 3.00 mm and reconstructed to 1.34 × 1.34 × 1.50 mm. The Z-axis field of view was 300 mm, and 110 slices with a thickness of 3 mm were acquired. The average scan time was approximately 4 min.

### 4D flow analysis: assessment of regurgitant fraction, pulmonary artery dilation, WSS, and blood flow velocity

**Fig. 2:**
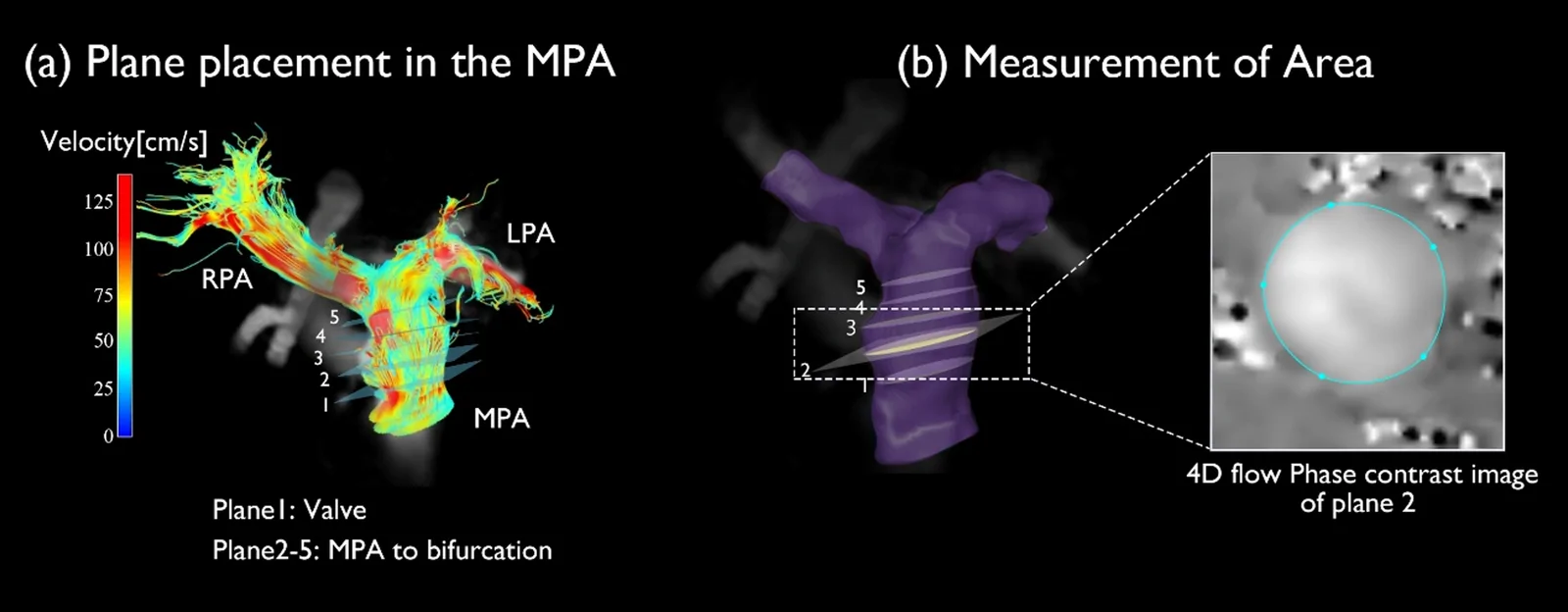
(**a**) Plane plancement in the MPA. Five measurement planes were positioned along the MPA. Plane 1 was placed at the valve level, and planes 2–5 were positioned at equal intervals from the MPA toward the bifurcation. (**b**) Measurement of the cross-sectional area. Phase-contrast images obtained at the predefined planes were manually contoured to measure the MPA cross-sectional area. The mean area of the five planes was used to evaluate the degree of dilation

4D flow analysis was performed using MR Caas 4D Flow (Philips Healthcare, Best, The Netherlands). Five cross-sectional planes were positioned from the valve to the main pulmonary artery (MPA) bifurcation on 4D flow data (Fig. 2a). Pre-TPVI, Plane 1 was set at the valve with reference to the sagittal cine MRI images, Planes 2–5 were set evenly up to just before the bifurcation of the MPA.

The regurgitant fraction derived from 4D flow (RF-4D) was measured at Plane 2, located approximately 10 mm distal to the valve. This position was selected to avoid flow disturbances caused by valve motion while ensuring an accurate measurement of the regurgitant flow into the right ventricle [11]. 

To assess the degree of pulmonary artery dilation pre-TPVI, cross-sectional areas were measured at 5 planes using 4D flow phase-contrast images during the systolic phase, and the average cross-sectional area of these 5 slices was used for analysis (Fig. 2b). The association between valvular enlargement and pulmonary artery dilation was evaluated using these five cross-sectional areas.

WSS (τ) is the tangential force exerted by blood flow on the endothelium, the innermost layer of the vascular wall. It is defined as the product of the axial shear rate of the vessel, which is calculated using the blood flow velocity (u), velocity gradient (du/dr) from the vessel wall, and blood viscosity (µ), which depends on hematocrit (Hct).

$$\:\tau\:=\mu\:\left(Hct\right)\frac{du}{dr}\:\:$$

**Fig. 3:**
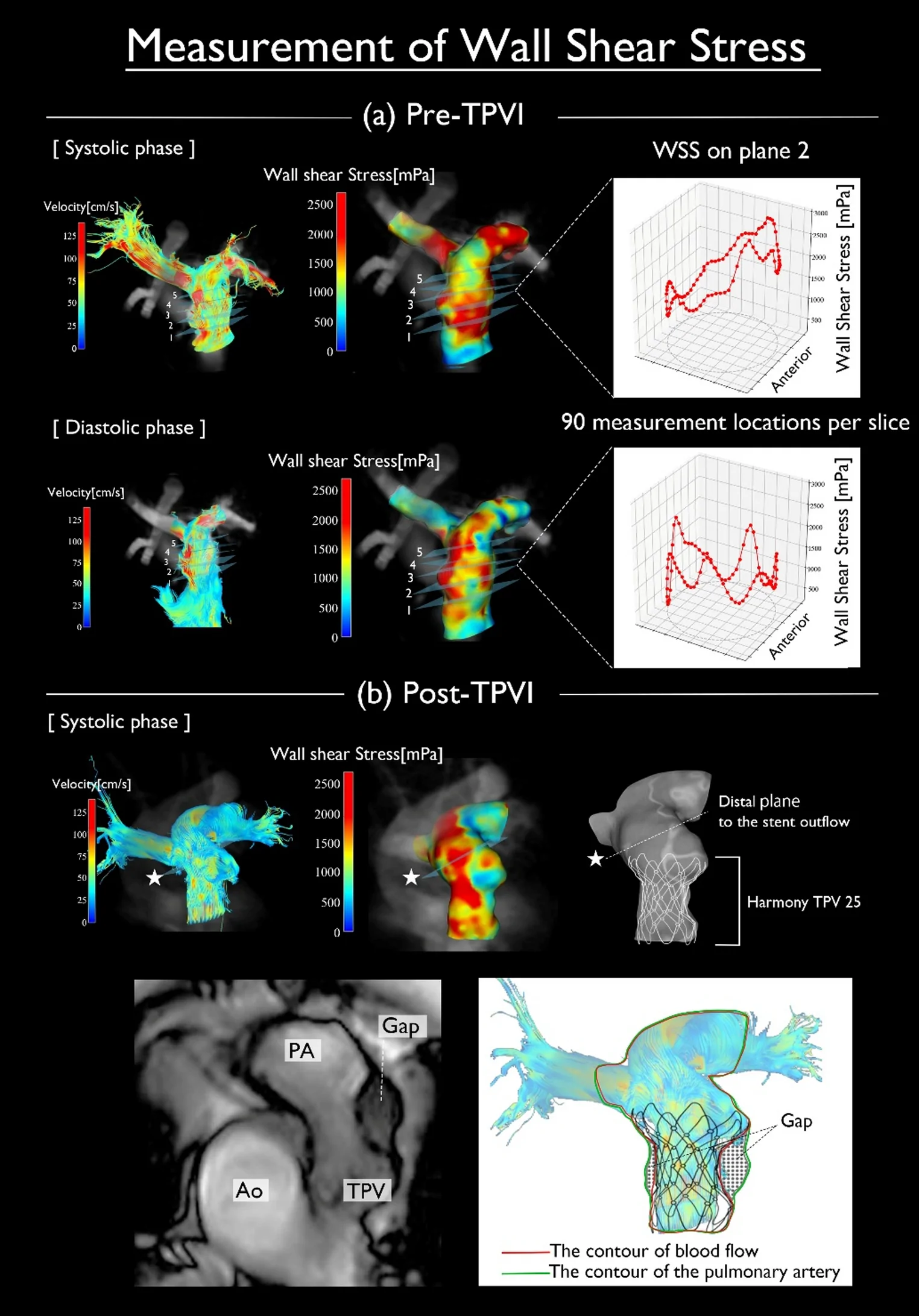
Measurement of WSS before and after TPVI using 4D flow MRI. (**a**) The right graph shows a three-dimensional visualization of WSS values at 90 sampling points. (**b**) The top panel shows blood flow images within the pulmonary artery post-TPVI, the 3D vessel model, WSS, and stent location. Post-TPVI, WSS was assessed at the most distal slice to minimize stent-related artifacts. The bottom panel presents a schematic diagram illustrating the Harmony TPV25 implanted in the MPA as imaged by CINE MRI, along with the corresponding blood flow image, stent, and pulmonary artery anatomy. Abbreviation; PA, pulmonary artery; Ao, aorta; TPV, Harmony TPV

Blood flow velocity was calculated based on the velocity profile within the pulmonary artery. In the Caas, the WSS was visualized through color mapping with a set threshold on a 3D vessel model created from 4D flow data reconstructed into a 3D volume. On cross-sectional slices of the 3D vessel model, the WSS values were recorded at 90 evenly spaced locations (Fig. 3a). For analysis, the average WSS across 5 cross-sectional slices was calculated, based on measurements taken at 10 phases of the cardiac cycle.

Post-TPVI, WSS was evaluated using only the most distal slice of the main pulmonary artery. The other four proximal slices were excluded from the analysis because they overlapped with the Harmony TPV 25 stent housing, making them susceptible to metal artifacts. Using the artifact-free distal slice ensured an accurate assessment of the hemodynamic impact on the native arterial wall (Fig. 3b).

For the assessment of blood flow velocity, the mean flow velocity per slice was calculated across 10 phases of the cardiac cycle.

### Statistical analysis

Changes in BNP, biventricular volumes (EDVi, ESVi), cardiac function (CO, EF), and regurgitant fraction (RF-4D) before and after TPVI were compared using paired t-tests.

The normality of data distribution was assessed using the Shapiro-Wilk test. For correlation analysis between preoperative variables and MPA cross-sectional area or WSS, Pearson’s correlation coefficient (r) was used for normally distributed variables, whereas Spearman’s rank correlation coefficient (rho) was employed for variables with a non-normal distribution to ensure statistical robustness.

In addition, the correlation between the cross-sectional area of the pulmonary valve and those of four planes (planes 2–5) of the main pulmonary artery was assessed using Pearson’s correlation coefficient.

To identify independent factors associated with MPA dilation, a multivariable linear regression analysis was performed. Based on the results of the univariate correlation analysis and clinical relevance, three variables—age, RF-4D, and diastolic WSS—were entered into the model to avoid overfitting given the sample size (*n* = 30). Multicollinearity was assessed using the variance inflation factor (VIF). The statistical analyses were performed using Python (version 3.13) with ths statsmodels library.

Interobserver reliability for RF-4D, MPA area, and WSS measurements was assessed using the intraclass correlation coefficient (ICC). The analysis was performed using the pingouin.intraclass_corr function in Python with Pingouin statistics package, employing a two-way mixed-effects model (ICC3) to evaluate consistency between raters.

A *p*-value < 0.05 was considered statistically significant.

## Results

### Study population and patient characteristics

A total of 30 patients with repaired TOF were finally included in this study. The patient characteristics, including age, sex, surgical history, and diagnostic right heart catheterization data (right ventricular systolic pressure [RVSP], right ventricular end diastolic pressure [RVEDP], mean pulmonary arterial pressure [PAP]) prior to TPVI are shown in Table 1. The mPAP was 18.5 ± 7.2 mmHg, with four patients exhibiting values above 25 mmHg. Of the surgical repairs, 25 involved a transannular patch, while none underwent the Rastelli procedure.

**Table 1:** Clinical and right heart catheterization data in 30 repaired tetralogy of Fallot patients

	All 30 patients
Age	45.7 ± 14.9
Male/Female	11/19
**Surgery**	
Transannular patch	25
Infundibular resection	5
valvotomy	3
**Right heart catheter**	
RVSP (mmHg)	41.5 ± 10.3
RVEDP (mmHg)	10.9 ± 4.3
mPAP (mmHg)	18.5 ± 7.2

RVSP, RV systolic pressure; RVEDP, RV end diastolic pressure; Mean PAP, mean pulmonary arterial pressure

### Biochemical and CMR data pre- and post-TPVI

The biochemical and CMR data were compared between pre- and 3 months post-TPVI in all 30 patients (Table 2).

**Table 2:** Clinical and CMR data pre-, 3 months post-TPVI

	Pre TPVI	Post TPVI(3month)	*p*-value
BNP(pg/ml)*	80.8	60.0	0.075
RF-4D (%)	41.8 ± 13.2	2.1 ± 4.2	< 0.001
RVEDVi (ml)	159.2 ± 38.7	107.7 ± 33.6	< 0.001
RVESVi (ml)	98.8 ± 34.0	79.1 ± 28.5	< 0.001
RVEF (%)	37.2 ± 11.7	26.8 ± 7.3	< 0.001
CO-RV (l/min)	3.8 ± 1.0	3.9 ± 0.9	0.881
LVEDVi (ml)	77.9 ± 15.8	83.3 ± 19.1	0.099
LVESVi (ml)	42.9 ± 13.6	42.2 ± 15.6	0.730
LVEF (%)	45.7 ± 10.2	50.5 ± 9.8	0.009
CO-LV (l/min)	3.6 ± 1.0	4.0 ± 1.0	0.016

BNP, brain natriuretic peptide; RF-4D, regurgitant fraction mesured by 4D Flow; RF-2D, regurgitant fraction mesured by 2D phase contrast; RVEDVi, RV end diastolic volume index; RVESVi, RV end systolic volume index; RVEF, RV ejection fraction; CO-RV, cardiac output-RV; LVEDVi, LV end diastolic volume index; LVESVi, LV end systolic volume index; LVEF, LV ejection fraction; CO-LV, cardiac output-LV. P-values were calculated using a paired t-test. *BNP is shown only as a average value because of the large variance

Following TPVI, there was a marked and significant reduction in RF-4D, indicating successful elimination of pulmonary regurgitation. No significant correlation was found between age and preoperative RF-4D. Regarding RV metrics, TPVI led to a significant decrease in RVEDVi, reflecting favorable reverse remodeling. Although RVEF showed a significant decrease post-procedurally. In contrast, LV function showed improvement, with a significant increase in LVEF observed after the intervention.

### Factors associated with pre-TPVI pulmonary artery dilation

**Fig. 4:**
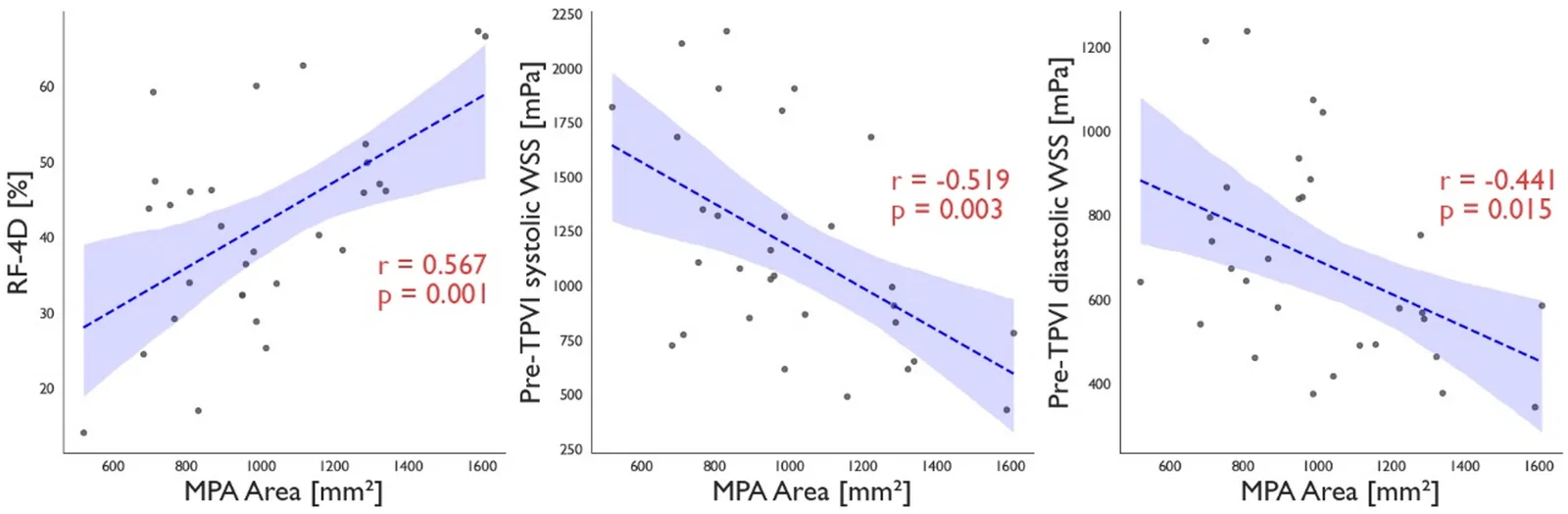
Scatter plots illustrate the relationships between the average cross-sectional area of the MPA and preoperative hemodynamic factors. (**A**) A strong positive correlation is observed between RF-4D. (**B**) A significant inverse correlation is shown between systolic WSS and MPA area. (**C**) Similarly, early diastolic WSS demonstrates a significant negative correlation with MPA area. Correlation coefficient values are Pearson’s correlation coefficients (r). Abbreviation; MPA, main pulmonary artery; RF-4D, the 4D flow-derived regurgitant fraction; TPVI, transcatheter pulmonary valve implantation; WSS, wall shear stress

The average cross-sectional area of the MPA pre-TPVI (1004.4 ± 265.5 mm^2^), derived from 5 slices measured by 4D flow, showed a strong positive correlation with preoperative RF-4D (Table 3; Fig. 4). In contrast, no significant correlation was observed with mPAP measured by right heart catheterization. A significant negative correlation was found with both systolic WSS and early diastolic WSS.

**Table 3:** Correlation between the average cross-sectional area of the main pulmonary artery [mm2] and clinical and CMR data

Pre-operative parameters	Correlation coefficient	*p*-value
Age	0.120	0.529
BNP (pg/ml)	−0.100^†^	0.599
RF-4D (%)	0.567	0.001
CO-RV (l/min)	−0.068	0.720
RVEDVi (ml)	0.264	0.158
RVESVi (ml)	0.210	0.265
RVEF (%)	0.086	0.652
CO-LV (l/min)	−0.068	0.720
LVEDVi (ml)	−0.523	0.003
LVESVi (ml)	−0.439	0.015
LVEF (%)	0.134	0.479
RVSP (mmHg)	0.080	0.673
RVEDP (mmHg)	−0.160^†^	0.398
mPAP (mmHg)	0.017^†^	0.927
Systolic mean velocity (cm/s)*	−0.623	< 0.001
Diastolic mean velocity (cm/s)*	−0.265	0.156
Systolic WSS (mPa)	−0.519	0.003
Diastolic WSS (mPa)	−0.441	0.015

*Mean velocity indicates the mean blood flow velocity of multiple slices measured in 4D flow. Correlation coefficient values are Pearson’s correlation coefficients (r) unless otherwise indexed. ^†^Spearman’s rank correlation coefficient (rho) was applied for variables with a non-normal distribution as assessed by the Shapiro-Wilk test

The pulmonary valve annulus area (964.2 ± 304.7 mm^2^) showed a strong correlation with the mean cross-sectional area of four other slices of the MPA from proximal to distal segments (1014.4 ± 272.7 mm^2^, *r* = 0.721, *p* < 0.001).

Multivariable linear regression analysis demonstrated that RF-4D (β = 11.52, *p* < 0.001) and diastolic WSS (β = −0.54, *p* = 0.003) were significantly and independently associated with MPA area, while age was not a significantly associated(*p* = 0.352). The overall model was significant (R^2^ = 0.525, *p* < 0.001). VIF analysis demonstrated no evidence of multicollinearity among the explanatory variables in either model (all VIF < 1.3).

### Analysis of WSS in the cardiac cycle

**Fig. 5:**
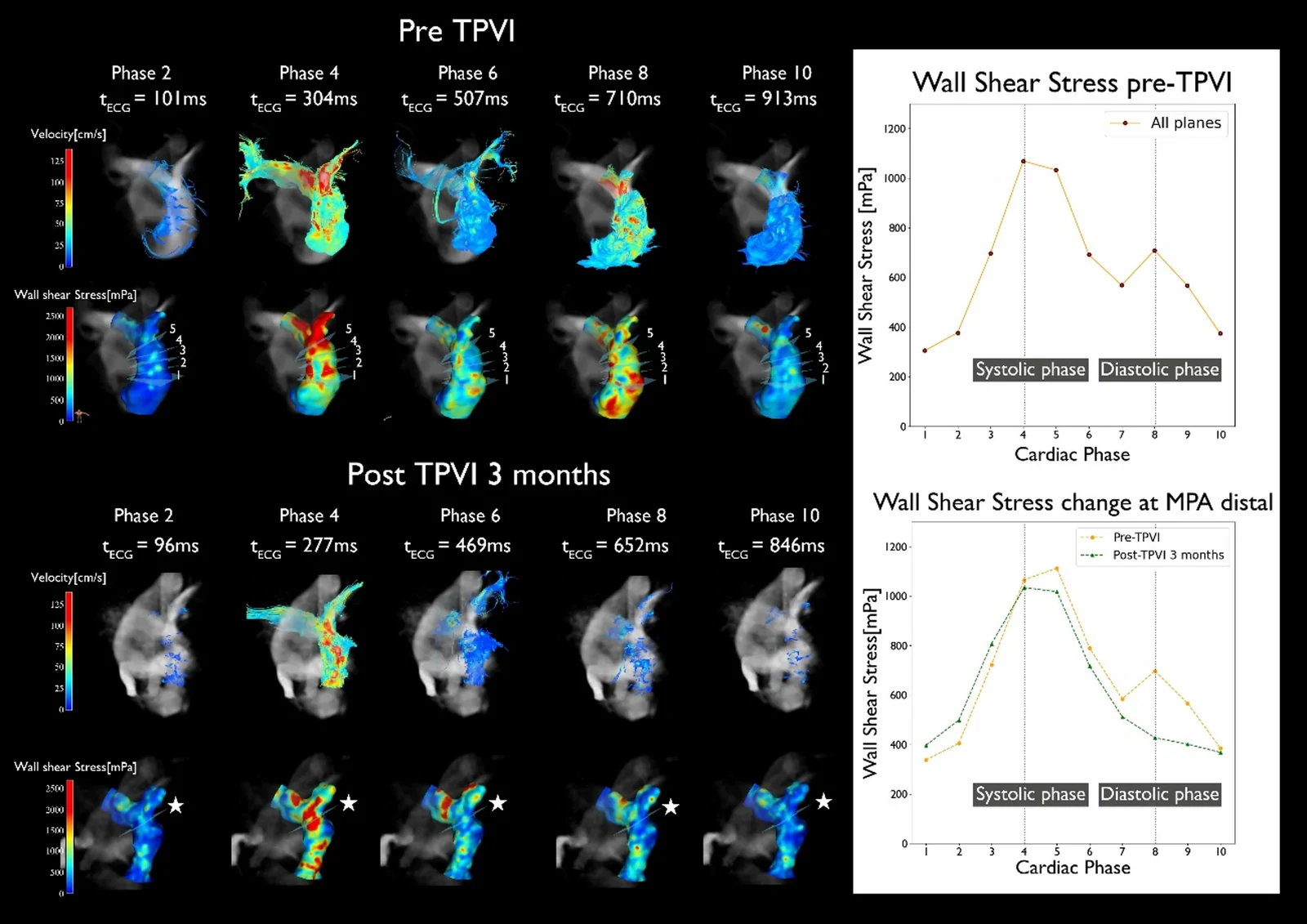
WSS analysis throughout the cardiac cycle before and after TPVI. On the left, representative blood flow velocity fields and WSS distributions in the pulmonary artery at selected cardiac phases are shown before TPVI (upper panels) and 3 months after TPVI (lower panels). On the right, statistical results from a cohort of 30 patients are presented: average WSS values across five MPA slices before TPVI (upper graph) and comparison between pre- and post-TPVI measurements at the most distal slice (Plane 5) (lower graph)

Visual observation of color-mapped WSS on the 3D vascular model confirmed that numerous patients in Pre-TPVI experienced high WSS in early diastole due to pulmonary regurgitation. The average WSS across 5 slices for each patient was calculated and graphed for each of the 10 phases per cardiac cycle. High WSS values were measured in the pulmonary artery not only in systole, but also in early diastole, showing a bimodal occurrence throughout the cardiac phase. (Fig. 5).

In systole, pre-TPVI mean WSS from the valve to the MPA bifurcation demonstrated a negative correlation with age (Table 4). A similar negative correlation with age was observed in early diastole. No significant association was found between WSS and RF-4D.

**Table 4:** Correlation of pre-TPVI systolic and early diastolic wall shear stress with pre-TPVI clinical and CMR data

Pre-TPVI	Systolic wall shear stress		Diastolic wall shear stress	
	Correlation coefficient	p-value	Correlation coefficient	p-value
Age	−0.484	0.007	−0.393	0.031
BNP (pg/ml)	−0.395†	0.031	−0.252^†^	0.18
RF-4D (%)	−0.301	0.105	−0.013	0.945
RVEDVi (ml)	−0.228	0.225	0.191	0.312
RVESVi (ml)	−0.335	0.07	0.078	0.681
RVEF (%)	0.212	0.261	0.045	0.813
CO-RV (l/min)	0.284	0.129	0.029	0.878
LVEDVi (ml)	0.468	0.009	0.568	0.001
LVESVi (ml)	0.282	0.131	0.297	0.111
LVEF %)	−0.053	0.779	0.025	0.897
CO-LV (l/min)	0.384	0.036	0.279	0.136
RVSP (mmHg)	−0.152	0.423	−0.028	0.883
RVEDP (mmHg)	−0.114†	0.549	−0.264†	0.159
mPAP (mmHg)	−0.293†	0.116	−0.075†	0.692

Correlation coefficient values are Pearson’s correlation coefficients (r) unless otherwise indexed. ^†^Spearman’s rank correlation coefficient (rho) was applied for variables with a non-normal distribution as assessed by the Shapiro-Wilk test

### Changes in WSS pre- and post-TPVI

The biphasic WSS observed pre-TPVI visually decreased in early diastole 3 months post-TPVI, becoming prominent only during systole (Fig. 5).

At 3 months post-TPVI, WSS was evaluated using the most distal slice (Plane 5). For pre- and post-procedural comparison, WSS was consistently assessed at this same location to ensure comparability. While systolic WSS showed no significant change compared to pre-procedural values (1122.7 ± 465.2 mPa vs. 1035.5 ± 460.5 mPa, *p* = 0.630), early diastolic WSS exhibited a significant reduction (678.5 ± 269.7 mPa vs. 439.4 ± 149.7 mPa, *p* < 0.001).

### Reproducibility

Intraclass correlation coefficients demonstrated high interobservers’ agreement between the two raters across all measured parameters. The ICC values with 95% confidence intervals (CI) were as follows: RF-4D = 0.916 (95% CI: 0.78–0.97), Area = 0.849 (95% CI: 0.62–0.95), systolic WSS = 0.928 (95% CI: 0.80–0.98), and early diastolic WSS = 0.932 (95% CI: 0.81–0.98).

## Discussions

### In vivo evaluation of WSS and its biological significance

To the best of our knowledge, this is the first study to evaluate WSS before and after TPVI. A key consideration in preoperative WSS evaluation is the presence of a biphasic pattern of WSS during the cardiac cycle, with high WSS occurring during early diastole due to pulmonary artery regurgitation. Post- TPVI, early diastolic WSS decreased with improvement of pulmonary artery regurgitation.

Vascular remodeling in the pulmonary circulation, a low-pressure system, is governed by mechanotransduction processes that may differ from those established in high-pressure systemic arteries. In the systemic artery, low WSS is a well-recognized driver of intimal thickening and atherosclerotic plaque formation [6, 7], whereas high WSS may trigger the production of vasodilators, such as prostaglandin I2 (PGI2) and nitric oxide (NO). Previous studies have indicated that WSS modulations influence PGI2 formation and oxidative stress pathways, with high WSS promoting vascular smooth muscle relaxation [12–15].

While the precise role of WSS in the pulmonary vascular bed remains to be fully elucidated, insights from other low-pressure environments, such as early vein graft models, suggest that WSS is a critical regulator of vessel caliber through distinct mechanisms [8]. In these models, low shear stress promotes inward remodeling and wall thickening, whereas high shear stress has been shown to induce initial intimal changes followed by a predominant outward expansion [8]. This outward remodeling is thought to be an adaptive response aimed at reducing the elevated shear stress back toward homeostatic levels [6, 8]. Blood vessels are thought to remodel in response to shear stress to maintain distinct “setpoints,“ [16, 17] with differential VEGFR3 expression influencing these setpoints across arteries, veins, and lymphatic vessels [18]. 

In the context of repaired TOF, the MPA is chronically exposed to elevated diastolic WSS driven by pulmonary regurgitation. While caution is required when applying these systemic findings directly to the pulmonary artery, these biological responses likely contribute to the vascular dilation observed in our cohort, supporting the role of elevated diastolic WSS as a key stimulus for MPA expansion.

### Flow-related mechanical stress as a primary driver of pulmonary artery remodeling

Both WSS and pressure overload are known to contribute to vasodilation [19]. However, in adult patients post-tetralogy of Fallot repair, RF-4D demonstrated a strong positive correlation with the mean pulmonary artery cross-sectional area, whereas its correlation with mPAP was weak (Table 3). These findings suggest that flow-related factors mediated by regurgitant flow, rather than pressure overload, may play a more important role in pulmonary artery dilatation. However, in our cohort, the number of patients with elevated pulmonary artery pressure (mPAP > 25 mmHg) was limited, and thus caution is required when interpreting the lack of association between pressure load and pulmonary artery size.

It is important to acknowledge that other hemodynamic mechanisms can also drive MPA remodeling. For instance, post-stenotic dilation is a well-recognized phenomenon in patients with pulmonary valve stenosis; in such cases, accelerated systolic flow and the resulting increase in systolic WSS are thought to contribute to vascular expansion [20]. 

### Relationship between pulmonary artery dilation and pulmonary regurgitation progression

The present study demonstrated that even in patients with TOF, whose pulmonary arteries often exhibit complex morphologies [21], pulmonary valve annulus size strongly correlated with the average MPA dilatation. When integrated with previous reports indicating that annulus enlargement exacerbates pulmonary regurgitation [22], our findings suggest a complex interplay between morphological and functional deterioration.

Given that the majority of our cohort underwent transannular patch repair, the initial impairment of valve function likely serves as a primary trigger for PR. This, in turn, may initiate a vicious cycle: chronic PR leads to progressive MPA dilation, which further enlarges the pulmonary annulus, thereby worsening the regurgitant volume.

### Clinical relevance of WSS measurement

In patients with repaired TOF, WSS measurement may provide a means of assessing the activity of pulmonary artery remodeling.

The inverse correlation observed between diastolic WSS and the degree of MPA dilation likely reflects a homeostatic vascular response to maintain shear stress setpoints. In our multivariable analysis, diastolic WSS remained significantly associated with MPA area even after adjusting for the regurgitant fraction (RF-4D). This finding suggests that for a given volume of pulmonary regurgitation, a more severely dilated MPA exhibits lower diastolic WSS. This implies that WSS could serve as a hemodynamic marker to evaluate the current stage or “activity” of vascular remodeling in individual patients [23, 24].

Consequently, WSS has the potential as a parameter for classifying patients who are prone to progressive pulmonary regurgitation from those who are not. In this study, WSS derived from pulmonary regurgitation showed an inverse correlation with age, indicating that younger patients tended to exhibit higher WSS levels. This finding suggests that hemodynamic stress associated with pulmonary regurgitation may be relatively more pronounced in younger patients, which could correspond to an earlier or more dynamic phase of vascular remodeling.

In patients with repaired TOF, a clinical course can be hypothesized where worsening valve function over time leads to increased pulmonary regurgitation, triggering MPA dilation as a response to elevated WSS. As this remodeling process reaches its advanced stages, the WSS may eventually decrease due to the significant increase in vessel radius. This hypothesis should be interpreted with caution, and longitudinal studies are required to determine whether WSS can predict future disease progression.

### Changes in ventricular function before and after TPVI

In this study, TPVI resulted in a significant reduction in pulmonary regurgitation, accompanied by marked right ventricular (RV) reverse remodeling, as evidenced by a decrease in RVEDVi.

Despite this favorable structural change, RVEF decreased after TPVI. This finding can be partly explained by altered loading conditions, particularly reduced preload following elimination of regurgitant flow, as well as persistent regional wall motion abnormalities in the right ventricular outflow tract.

Importantly, conventional (non-corrected) RVEF may overestimate pre-intervention RV function because it includes regurgitant flow. A meta-analysis including 3,118 patients undergoing pulmonary valve replacement demonstrated that changes in non-corrected RVEF are variable and do not consistently show improvement after intervention [25]. Therefore, the observed decrease in RVEF in our study is not inconsistent with prior literature.

### Limitations

There are several limitations to this study. First, this was a single-center study with a relatively small cohort of 30 patients. In addition, although both pre- and post-procedural assessments were performed, the follow-up period was relatively short, limiting evaluation of long-term changes in WSS and vascular remodeling.

Second, the study population mainly consisted of patients with repaired tetralogy of Fallot and significant pulmonary regurgitation. Therefore, the findings may not be generalizable to other causes of pulmonary artery dilation, such as pulmonary stenosis or pressure overload. In addition, only a limited number of patients had elevated pulmonary artery pressure (mPAP > 25 mmHg), which may have reduced the ability to detect its association with pulmonary artery size.

Furthermore, the use of the Harmony TPV25 posed a limitation: the stent is designed with an outflow diameter of 43 mm—larger than the preoperative pulmonary artery—to prevent migration into the right ventricle after implantation. Therefore, it was not possible to evaluate whether improvements in diastolic WSS were associated with reductions in pulmonary artery dilation.

Finally, although our data suggest an association between WSS and pulmonary artery remodeling, the observational nature of this study precludes causal inference. Further longitudinal studies with long-term follow-up are required to clarify the causal relationship between hemodynamic stress and vascular structural changes over time.

## Conclusions

In adult patients with repaired tetralogy of Fallot, pulmonary artery dilation was more strongly associated with pulmonary regurgitation-derived flow metrics than with pulmonary artery pressure, suggesting a predominant role of flow-related hemodynamic stress in vascular remodeling. Diastolic WSS decreased following TPVI in parallel with improvement in pulmonary regurgitation, indicating its dynamic responsiveness to changes in loading conditions. WSS was independently associated with pulmonary artery dilation and showed an inverse relationship with age, suggesting its potential to reflect the stage of vascular remodeling.

These findings suggest that WSS may serve as a mechanistic marker of pulmonary artery remodeling in repaired TOF and may provide incremental value for risk stratification of patients with progressive pulmonary regurgitation and pulmonary artery dilation. Further longitudinal studies are needed to confirm its prognostic utility.

## Data Availability

The datasets generated or analyzed during the study are available from the corresponding author on reasonable request.
